# Artificial Intelligence Algorithms in Predictive Factors for Hematologic Toxicities During Concurrent Chemoradiation for Cervical Cancer

**DOI:** 10.7759/cureus.70665

**Published:** 2024-10-01

**Authors:** Ion Petre, Serban Negru, Radu Dragomir, Anca Bordianu, Izabella Petre, Luciana Marc, Daliborca Cristina Vlad

**Affiliations:** 1 Department of Biostatistics, Victor Babes University of Medicine and Pharmacy, Timisoara, ROU; 2 Department of Functional Science, Medical Informatics and Biostatistics, Victor Babes University of Medicine and Pharmacy, Timisoara, ROU; 3 Department of Medical Oncology, Oncohelp Oncology Center, Timisoara, ROU; 4 Department of Oncology, Victor Babes University of Medicine and Pharmacy, Timisoara, ROU; 5 Department of Obstetrics and Gynecology, Victor Babes University of Medicine and Pharmacy, Timisoara, ROU; 6 Department of Plastic and Reconstructive, Bagdasar-Arseni Emergency Hospital, Carol Davila University of Medicine and Pharmacy, Bucharest, ROU; 7 Department of Obstetrics and Gynecology, Pius Brinzeu Emergency County Clinical Hospital, Timisoara, ROU; 8 Department of Internal Medicine, Victor Babes University of Medicine and Pharmacy, Timisoara, ROU; 9 Department of Pharmacology, Victor Babes University of Medicine and Pharmacy, Timisoara, ROU; 10 Department of Laboratory Medicine, Pius Brinzeu Emergency County Clinical Hospital, Timisoara, ROU

**Keywords:** cervical cancer, concurrent chemoradiation, hematologic toxicities, hpv, stage iii

## Abstract

The most recent research conducted for the International Federation of Gynecology and Obstetrics indicates that, depending on the stage of cervical cancer (CC), several therapies can provide similar overall survival and progression-free survival rates. To determine the hematologic toxicities during concurrent chemotherapy for cervical cancer, we evaluated these two therapies (cisplatin or carboplatin). Hematologic markers have been studied using statistical models and descriptive statistics. Artificial intelligence models were built using the treatment data and all the information gathered from each patient after one or more administrations to forecast the CC stage. The information was gathered from stage III cervical cancer patients and provided by Oncohelp Hospital from the West Region of Romania. Many traditional machine learning techniques, such as naïve Bayes (NB), random forest (RF), decision trees (DTs), and a trained transformer called TabPFN, were used in the current study to obtain the tabular data. The algorithms NB, RF, and DTs yielded the greatest classification score of 100% when it came to cervical cancer prediction. On the other hand, TabPFN demonstrated an accuracy of 88%. The effectiveness of the models was evaluated by computing the computational complexity of traditional machine learning methods. Early detection increases the likelihood of a good prognosis during the precancerous and malignant stages. Being aware of any indications and symptoms of cervical cancer can also help to prevent delays in diagnosis. These hematologic toxicities, which have been demonstrated to grow linearly with lowering hematologic markers below their normal expectations, would significantly impair patients' quality of life.

## Introduction

Adverse effects on the blood system, or hematotoxicities, are frequently observed after concurrent chemoradiation therapy for cervical cancer [1]. Although there are many potential predictors of these toxicities, a few of them have been noted in the literature [2-5]. It is crucial to remember that each person may react differently to therapy, and it is not always easy to forecast toxicities [6]. Nonetheless, the few typical determinants are as follows: chemotherapy regimen, dose and schedule of chemotherapy, baseline hematologic parameters, bone marrow irradiation, age and performance status, comorbidities, genetic factors, nutritional status, use of growth factors, and treatment interruptions [7-9].

Negative effects on the blood and bone marrow, known as hematologic toxicities, are usually caused by medical procedures like chemotherapy, radiation therapy, or the administration of prescription drugs. Several clinical disorders may result from these toxicities' effects on blood cell formation, lifespan, and function. The primary hematologic toxicities include anemia and pancytopenia. 

The chance and severity of hematologic toxicities can be affected by particular chemotherapy medications. Treatment for cervical cancer frequently involves platinum-based chemotherapy, such as cisplatin, which may have increased hematopoietic function [10,11].

The discovery of cisplatin served as a catalyst for the increased interest in compounds containing platinum and other metals as possible anticancer medications. When cisplatin was used clinically, it was found that many patients with a variety of malignancies, including blood vessels, soft tissue, bone, and sarcoma tumors, responded well to treatment. Today, the prognosis for such malignancies is improved [10].

Hematologic toxicity can be affected by chemotherapy dosage and delivery frequency. The risks of this may rise with higher dosages and more frequent intake. Toxicities may be more likely to occur in patients with pre-existing hematologic disorders or lower concentrations of baseline blood cells. Hematologic toxicity during pelvic radiation treatment may be influenced by the degree of bone marrow irradiation [12]. Higher radiation doses in regions rich in bone marrow may raise this risk. Hematologic toxicity may be more common in older patients or those with lower performance. The likelihood of hematologic toxicities may be elevated by the presence of other medical disorders, particularly those that impact the immune system or blood [13-18]. Genetic differences within an individual may impact treatment response and medication metabolism, thereby increasing the risk of hematologic toxicities [19-21]. Radiation and chemotherapy can have a worse effect on the hematopoietic system if there is malnutrition or low nutritional status [22]. Hematopoietic growth factors, such as granulocyte colony-stimulating factor (G-CSF), can be used prophylactically to reduce hematologic toxicities. However, a patient's unique circumstances and particular treatment plans will determine how they are used. Any treatment pauses or delays might affect the toxicity profile and overall efficacy. Keeping to the recommended treatment plan is crucial.

Medical professionals must evaluate these parameters both before and during therapy to appropriately anticipate and manage hematologic toxicities. Close observation and customized treatment programs can help maximize positive patient outcomes and reduce negative consequences. Furthermore, improvements in personalized medicine and supportive care also contribute to lessening the effects of toxicities during cancer therapy [23-26].

Ensuring the efficacy of the therapeutic strategy while minimizing treatment-related side effects is the basic goal of controlling hematologic toxicities during concurrent chemoradiation for cervical cancer. Key goals include the prevention and mitigation of hematologic toxicities, identification of high-risk patients, optimization of chemotherapy regimens, individualized treatment plans, regular monitoring of hematologic parameters, use of supportive care measures, patient education and communication, timely intervention and dose modifications, management of treatment interruptions, research and clinical trials, quality of life considerations, and multidisciplinary collaboration [27-31].

A routine is set up for routine blood count monitoring both during and after treatment to quickly identify and address any potential hematologic toxicities [32].

The purpose of utilizing supportive care techniques is to treat and mitigate hematologic toxicities; these techniques include blood transfusions and the use of hematopoietic growth factors (e.g., G-CSF). In addition, it is important to inform patients of the symptoms of any possible hematologic toxicities, as well as the significance of reporting any abnormal blood count symptoms as soon as possible. An advantage is to offer encouragement to the patients and healthcare professionals to communicate openly. For better results, it is necessary to respond to hematologic toxicities promptly by implementing dosage changes, stopping treatments, or modifying chemotherapy and radiation regimens [29].

The purpose of defining procedures for handling treatment interruptions is so that patients can receive the supportive care they need during these times and go back to their treatments as soon as it is feasible. Another aspect is the promotion of and participation in studies and clinical trials that seek to find new ways to treat patients that have less hematologic toxicity without sacrificing therapeutic efficacy [6]. The significance of striking a balance between treatment efficacy and minimizing the impact on a patient's everyday life and well-being by including quality-of-life evaluations into the overall treatment plan plays an important role in reducing the negative effects. It is crucial to ensure cooperation between radiation oncologists, hematologists, oncologists, and other medical specialists to optimize the overall treatment of patients with cervical cancer who are receiving concurrent chemoradiation [27].

To achieve the best possible treatment results for cervical cancer patients, medical professionals want to improve the safety and tolerability of concurrent chemoradiation [33,34].

Both carboplatin and cisplatin are platinum-based chemotherapy medications that are often used to treat different types of cancer [35-41]. Despite being in the same drug class, cisplatin and carboplatin differ in a few ways, including their pharmacology, adverse effects, and therapeutic uses.

Several variables, such as the particular kind of cancer being treated, the patient's general health, and the ideal ratio of toxicity to efficacy, will determine which of the two drugs is used. A patient's unique qualities and the intended course of therapy may determine which is better in a given situation [42,43].

This work attempts to understand the factors for predicting hematologic toxicities during concurrent chemoradiation for CC to find proper treatment and reduce side effects. The following is a summary of the paper's organization. The information and techniques adhere to the CC guidelines outlined in Section 2. Section 3 of the results shows the investigation flow for CC. Section 4 presents a relevant discussion on the topic. Section 5 summarizes the research and its findings.

## Materials and methods

The clinical characteristics of patients with cervical cancer who had various therapies (carboplatin or cisplatin) according to their stage and other medical histories were the subject of data collection, which took place between 2018 and 2023. Only patients who completed an informed consent form and provided all necessary data were included in the study. Oncohelp Hospital from Timisoara ensured the availability of the data.

More variables, like age, weight, body area, histopathological exam, stage, treatment, and hepatological markers (neutrophile, leucocyte, lymphocyte, hemoglobin), and creatinine, were recorded after administration in each treatment session based on the patient’s stage of cervical cancer and personal needs. 

This research approach takes into account the inter- and transdisciplinary techniques needed to address this difficult problem, combining studies, descriptive statistics, artificial intelligence analysis, and prediction. These techniques include quantitative and qualitative methods for classifying a patient’s disease stage based on their therapy profiles. 

To conduct the statistical analysis, the Jasp program (version 2023) was utilized. The impact of the therapy that was employed to treat the patients was examined via descriptive statistics. The threshold value for significance that we employed was α=0.05.

Artificial intelligence algorithms

**Figure 1:**
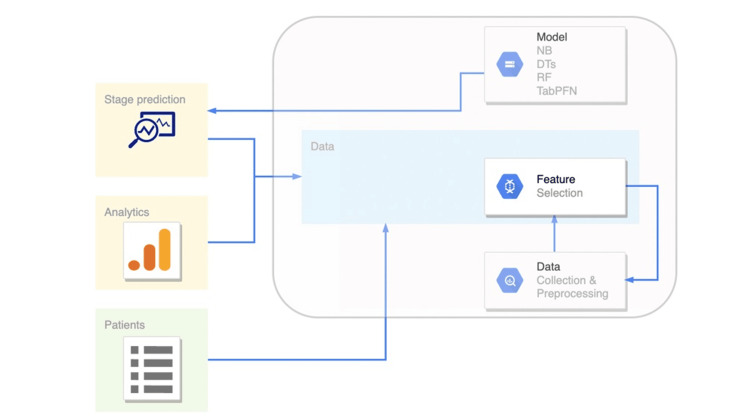
The architecture

As methods, we used four algorithms to classify patients’ disease stages based on their profiles. In Figure 1, we depict the architecture proposed to detect the evolution of hepatological and creatinine levels in combating cervical cancer through cisplatin or carboplatin administration. Popular machine learning (ML) algorithms used for classification problems include random forest, decision trees, naïve Bayes, and TabPFN. The probabilistic model, known as naïve Bayes, is predicated based on the feature independence assumption and Bayes' theorem. Although decision trees offer a simple approach to simulated decision-making procedures, overfitting may occur. Because a random forest is an ensemble of decision trees, it minimizes the shortcomings of individual trees while utilizing the advantages of several models to increase accuracy and generalizability. The particulars of the dataset and the objectives of the current assignment determine which approach is best. With minimal need for hyperparameter adjustment, TabPFN is a trained transformer that can perform supervised classification for tiny tabular datasets in under a second, making it comparable with cutting-edge classification techniques. The TabPFN algorithm, designed for robust performance in scenarios with limited data, was employed due to the insufficiency of the dataset available.

## Results

Treatment effectiveness analysis shows how the patient's stage is affected by treatment options (carboplatin or cisplatin)-both in terms of decreasing and halting the progression of the illness. AI modeling techniques were used to conduct this.

Data analysis and statistics

Descriptive statistics for a cohort of cancer patients (n=45), categorized by age and cancer stage (stage III), reveal clinical insights (Table 1). The mean age of patients is 54 years, with a standard deviation of 11 years, indicating a relatively older patient population. The age range spans from 35 to 73 years. Regarding cancer stage, 6.67% of the patients are diagnosed at stage IIIA, 37.8% at stage IIIB, 48.89% at stage IIIC1, and 6.67% at stage IIIC2. The median age at diagnosis tends to increase with cancer stage; stage IIIA patients have a median age of 67, stage IIIB 56, stage IIIC1 54, and stage IIIC2 64, suggesting that more advanced stages are more common in older individuals. These statistics highlight the need for age-specific strategies in cancer management and the importance of early detection, particularly in older populations.

**Table 1:** Descriptive statistics according to age and cancer stage Cervical cancer stages: IIIA, IIIB, IIIC1, and IIIC2 The data has been represented as mode, median, mean, std. deviation, Shapiro-Wilk, p-value of Shapiro-Wilk, minimum and maximum

Age				
	IIIA	IIIB	IIIC1	IIIC2
Valid	3	17	22	3
Missing	0	0	0	0
Mode	65.00	57.00	38.00	35.00
Median	67.00	56.00	54.00	64.00
Mean	67.33	54.94	53.86	54.66
Std. Deviation	2.517	10.371	11.124	17.039
Shapiro–Wilk	0.987	0.971	0.954	0.775
P-value of Shapiro–Wilk	0.780	0.840	0.378	0.056
Minimum	65.00	38.00	36.00	35.00
Maximum	70.00	73.00	72.00	65.00
ᵃ More than one mode exists; only the first is reported.				

**Figure 2:**
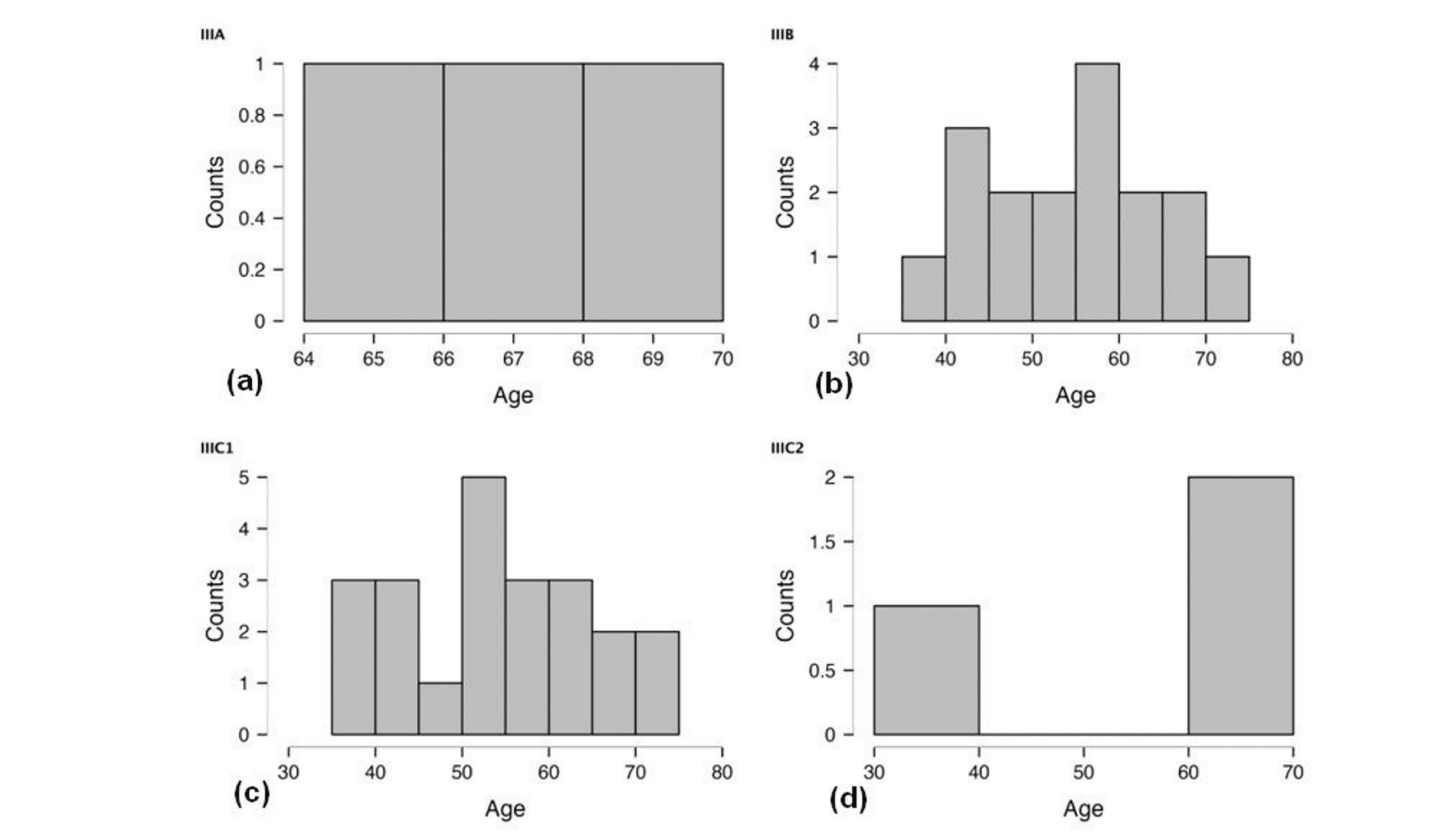
Cervical cancer case distribution by age (a) Stage III A cervical cancer distribution by age, (b) Stage III B cervical cancer distribution by age, (c) Stage III C1 cervical cancer distribution by age, (d) Stage III C2 cervical cancer distribution by age

Cervical cancer is not as frequent in younger women (under 30); however, it may occur at any age. Extended infection with high-risk variants of the human papillomavirus (HPV) is frequently linked to cervical cancer [44-47]. Since the immune system frequently eradicates HPV infections in younger age groups, there is a lower chance of developing cervical cancer. Cervical cancer incidence rises with age, with women in their 40s and 60s often having the greatest rates (Figure 2). In order to identify and treat precancerous alterations early on in the course of these years, routine screening, such as Pap smears or HPV tests, is essential. Older age groups may have a lower risk of cervical cancer, particularly if they have had lifelong checkups. On the other hand, women who have chronic HPV infections or have not had routine testing remain vulnerable [48-50].

**Figure 3:**
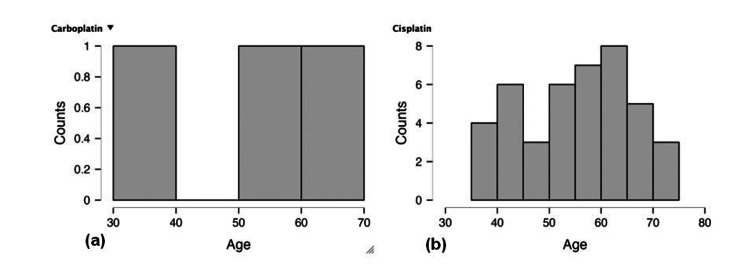
Cervical cancer case distribution by therapy (carboplatin or cisplatin) (a) Carboplatin therapy, (b) Cisplatin therapy

Various criteria, including the patient's health status, the stage of cervical cancer, and the possible adverse effects of the drugs, influence the decision to use either carboplatin or cisplatin alone or in combination [51,52]. Figure 3 shows that the most popular treatment was based on cisplatin, which covered a wider age range than carboplatin. Due to limited data, the study focuses exclusively on stage III cancer patients, comprising a total of 45 individuals. Within this cohort, 42 patients are receiving cisplatin treatment, while the remaining three are being treated with carboplatin. This distribution allows for a comparative analysis of the efficacy and side effects of these two commonly used platinum-based chemotherapeutic agents in a stage III cancer setting.

In the treatment of stage III cervical cancer, the administration of platinum-based chemotherapeutic agents like cisplatin and carboplatin is common. The dosage of these drugs is often calculated based on the patient's body surface area (BSA) to tailor the treatment to the individual's size and ensure efficacy while minimizing toxicity. The mean body area upon the administration of platinum-based chemotherapeutic agents like cisplatin and carboplatin tends to increase with cisplatin; carboplatin patients have a mean body area of 1.688, and cisplatin patients 1.696 (Table 2).

**Table 2:** Body area regarding therapy (Carboplatin vs. Cisplatin) The data has been represented as mean, std. deviation, minimum and maximum

	Body area	
	Carboplatin	Cisplatin
Valid	3	42
Missing	0	0
Mean	1.688	1.696
Std. Deviation	0.175	0.328
Minimum	1.488	0.000
Maximum	1.812	2.159

To determine whether there are any statistically significant differences between the means of the groups with varying stages (IIIA, IIIB, IIIC1, and IIIC2), we used the ANOVA test (Table 3).

**Table 3:** ANOVA by stage Statistical significance (p-value < 0.05)

Variable	Cases	Sum of Squares	df	Mean Square	F	P
Lym1	Stage	5.77	3	1.92	3.94	0.015
	Residuals	19.97	41	0.48		
Tro1	Stage	67769.55	3	22589.85	3.97	0.014
	Residuals	233157.68	41	5686.77		
Hmg2	Stage	21.46	3	7.15	3.07	0.038
	Residuals	95.50	41	2.32		
Hmg3	Stage	39.55	3	13.18	5.94	0.002
	Residuals	86.49	39	2.21		
Hmg4	Stage	37.16	3	12.38	5.68	0.003
	Residuals	80.67	37	2.18		
Lym5	Stage	0.72	3	0.24	2.85	0.052
	Residuals	2.79	33	0.08		
Hmg5	Stage	30.07	3	10.02	3.39	0.029
	Residuals	97.43	33	2.95		
	Note: Type III sum of squares.					

In this study, key hematologic and renal markers, lymphocytes, neutrophils, leukocytes, creatinine, thrombocytes, and hemoglobin, were measured after each administration of carboplatin or cisplatin in the 45 stage III cancer patients, e.g., lymphocytes corresponding to Lym 1 to Lym 5. Monitoring these markers helped assess the impact of the treatments on the patients' immune function, blood cell counts, and kidney function. Regular evaluation of these parameters is crucial for the early detection of potential toxicities, allowing for timely interventions to manage adverse effects and optimize the therapeutic outcomes for both carboplatin and cisplatin regimens.

It was observed that there was statistical significance (p-value < 0.05) in the lymphocytes following the initial and final treatments (Lym1, Lym5), as well as in the thrombocytes after initial therapy (Tro1). The hemoglobin level was the most affected; we can observe that it presented statistical significance at the end of each treatment process (Hmg2, Hmg3, Hmg4, Hmg5). Patients were advised to adhere to a full dose of treatment for the lymphocytes that showed statistical significance only after the fifth and final treatment (Lym1, Lym5) [53-55].

The statistical significance of Tro2 and Tro4 emerged while examining the independent variables in the dataset based on the stage of cervical cancer (Table 4). These markers showed notable differences across the treatment administration, suggesting their potential role in the progression or severity of cervical cancer.

**Table 4:** Independent samples T-test The Brown–Forsythe test shows significance (p < .05), suggesting a violation of the equal variance assumption.

	T	Df	P
Age	-0.303	43	0.764
Weight	-0.163	42	0.871
Height	-0.929	42	0.358
Body area	-0.431	42	0.669
Neu1	-0.980	43	0.332
Leu1	-1.054	43	0.298
Lym1	-0.231	43	0.819
Tro1	-0.812	43	0.421
Hmg1	-0.017	43	0.987
Cre1	0.135	43	0.893
Neu2	-0.293	43	0.771
Leu2	-0.085	43	0.933
Lym2	0.628	43	0.534
Tro2	3.429	43	0.001
Hmg2	-0.828	43	0.412
Cre2	0.327	41	0.745
Neu3	0.738	41	0.465
Leu3	1.096	41	0.280
Lym3	1.249	41	0.219
Tro3	-0.169	41	0.867
Hmg3	-1.045	41	0.302
Cre3	0.091	39	0.928
Neu4	1.196	39	0.239
Leu4	1.678	39	0.101
Lym4	1.465	39	0.151
Tro4	2.400	39	0.021
Hmg4	-0.655	39	0.516
Cre4	-0.520	36	0.606
Neu5	1.111	35	0.274
Leu5	1.369	35	0.180
Lym5	0.647	35	0.522
Tro5	0.606	35	0.549
Hmg5	-0.743	35	0.463
Cre5	-0.584	32	0.564
Note. Student's T-test.			

After using the paired samples T-test (Table 5), it was evident that the delivery of either carboplatin or cisplatin presents statistical significance (p-value < 0.05), in contrast to creatinine.

**Table 5:** Paired samples T-test Statistical significance (p-value < 0.05)

Measure 1	Measure 2	t	Df	P
Neu1	Neu2	4.93	44	< .001
Neu2	Neu3	2.98	42	0.005
Neu3	Neu4	3.92	40	< .001
Neu4	Neu5	0.90	36	0.369
Neu1	Neu5	7.11	36	< .001
Leu1	Leu2	5.75	44	< .001
Leu2	Leu3	3.78	42	< .001
Leu3	Leu4	4.20	40	< .001
Leu4	Leu5	1.14	36	0.258
Leu1	Leu5	7.88	36	< .001
Lym1	Lym2	6.91	44	< .001
Lym2	Lym3	5.30	42	< .001
Lym3	Lym4	3.87	40	< .001
Lym4	Lym5	0.38	36	0.705
Lym1	Lym5	7.79	36	< .001
Hmg1	Hmg2	3.88	44	< .001
Hmg2	Hmg3	2.96	42	0.005
Hmg3	Hmg4	2.42	40	0.020
Hmg4	Hmg5	3.51	36	0.001
Hmg1	Hmg5	7.64	36	< .001
Tro1	Tro2	3.49	44	0.001
Tro2	Tro3	3.50	42	0.001
Tro3	Tro4	0.74	40	0.463
Tro4	Tro5	-0.14	36	0.883
Tro1	Tro5	5.53	36	< .001
Cre1	Cre2	-0.66	42	0.512
Cre2	Cre3	-0.28	39	0.776
Cre3	Cre4	-0.07	35	0.941
Cre4	Cre5	-0.98	31	0.331
Cre1	Cre5	-1,64	33	0.109
Note: Student's T-test.				

**Figure 4:**
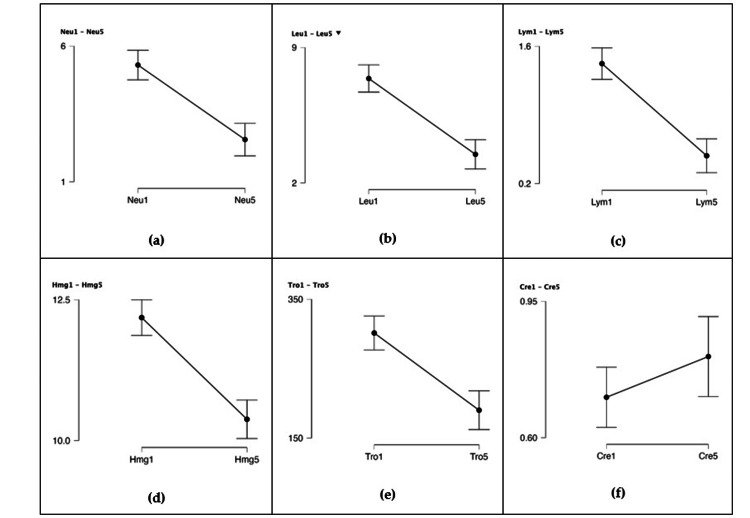
Hematological markers before and after therapy (carboplatin or cisplatin) corresponding to the dosage of the five treatments: (a) neutrophiles; (b) leucocytes; (c) lymphocytes; (d) hemoglobin; (e) thrombocytes; (f) creatinine

Table 6 illustrates that a subset of patients did not adhere to the whole five-treatment regimen (N = number of patients at each phase). The values for creatine and hematological markers (lymphocytes, leukocytes, hemoglobin, and thrombocytes) are accentuated, noting that leukocytes are affected and lymphocytes and hemoglobin are below the normal limit (Figure 4). Except for creatinine, which rose, all parameters typically declined when therapy was administered. The biological reference intervals for each of the examined markers are shown in Table 7 [56-60].

**Table 6:** Descriptives The data has been represented as N, Mean, SD, SE and Coefficient of variation

	N	Mean	SD	SE	Coefficient of variation
Neu1	45	5.30	2.16	0.32	0.409
Neu2	45	3.99	1.59	0.23	0.401
Neu3	43	3.44	1.21	0.18	0.351
Neu4	41	2.76	1.12	0.17	0.407
Neu5	37	2.55	1.31	0.21	0.513
Leu1	45	7.39	2.62	0.39	0.355
Leu2	45	5.45	1.87	0.27	0.344
Leu3	43	4.64	1.49	0.22	0.322
Leu4	41	3.80	1.35	0.21	0.356
Leu5	37	3.48	1.51	0.24	0.436
Lym1	45	1.42	0.76	0.11	0.538
Lym2	45	0.87	0.51	0.07	0.591
Lym3	43	0.64	0.39	0.06	0.608
Lym4	41	0.50	0.31	0.04	0.614
Lym5	37	0.48	0.31	0.05	0.646
Hmg1	45	12.18	1.66	0.24	0.137
Hmg2	45	11.68	1.63	0.24	0.139
Hmg3	43	11.30	1.73	0.26	0.153
Hmg4	41	11.02	1.71	0.26	0.156
Hmg5	37	10.37	1.88	0.30	0.181
Tro1	45	301.28	82.70	12.32	0.274
Tro2	45	247.91	93.37	13.92	0.377
Tro3	43	199.37	60.07	9.16	0.301
Tro4	41	191.39	63.91	9.98	0.334
Tro5	37	190.13	107.05	17.60	0.563
Cre1	45	0.70	0.21	0.03	0.303
Cre2	43	0.71	0.21	0.03	0.299
Cre3	41	0.72	0.25	0.04	0.356
Cre4	38	0.74	0.20	0.03	0.279
Cre5	34	0.80	0.50	0.08	0.622

**Table 7:** Biological reference interval (UM) Parameters analyzed were the following: neutrophile, leucocyte, lymphocyte, hemoglobin, thrombocyte, and creatinine

Parameter	Value
Neutrophile (Neu)	2-7 / 10^9^/L
Leucocyte (Leu)	3.98-10.04 / 10^9^/L
Lymphocyte (Lym)	3.93-5.22 / 10^12^/L
Hemoglobin (Hmg)	11.2-15.7 / g/dL
Thrombocyte (Tro)	150-450 / 10^9^/L
Creatinine (Cre)	0.51-0.95 / mg/dL

Artificial intelligence approach

In medical practice, it is becoming more and more common to use machine learning-based schemes to predict catastrophic illnesses, like cancer, in their early stages. One of the most common illnesses affecting women is cervical cancer, which may be prevented with an early diagnosis [61-65].

Because healthcare information is sensitive, there are several factors to consider while working with tabular medical data.

This section on predictive model selection (PMS) presents findings from studies utilizing many traditional machine learning techniques, such as NB, RF, DTs, and a trained transformer called TabPFN [66]. Although transformers are well recognized for their effectiveness in natural language processing (NLP) applications, they have also been modified for use with tabular data.

The algorithms naïve Bayes, random forest, and decision trees yielded the greatest classification score of 100% when it came to cervical cancer prediction. On the other hand, TabPFN demonstrated an accuracy of 88%. The effectiveness of the models is evaluated by determining the computational complexity of traditional machine learning methods. There is an 80% train dataset and a 20% validation dataset in the tabular data.

**Figure 5:**
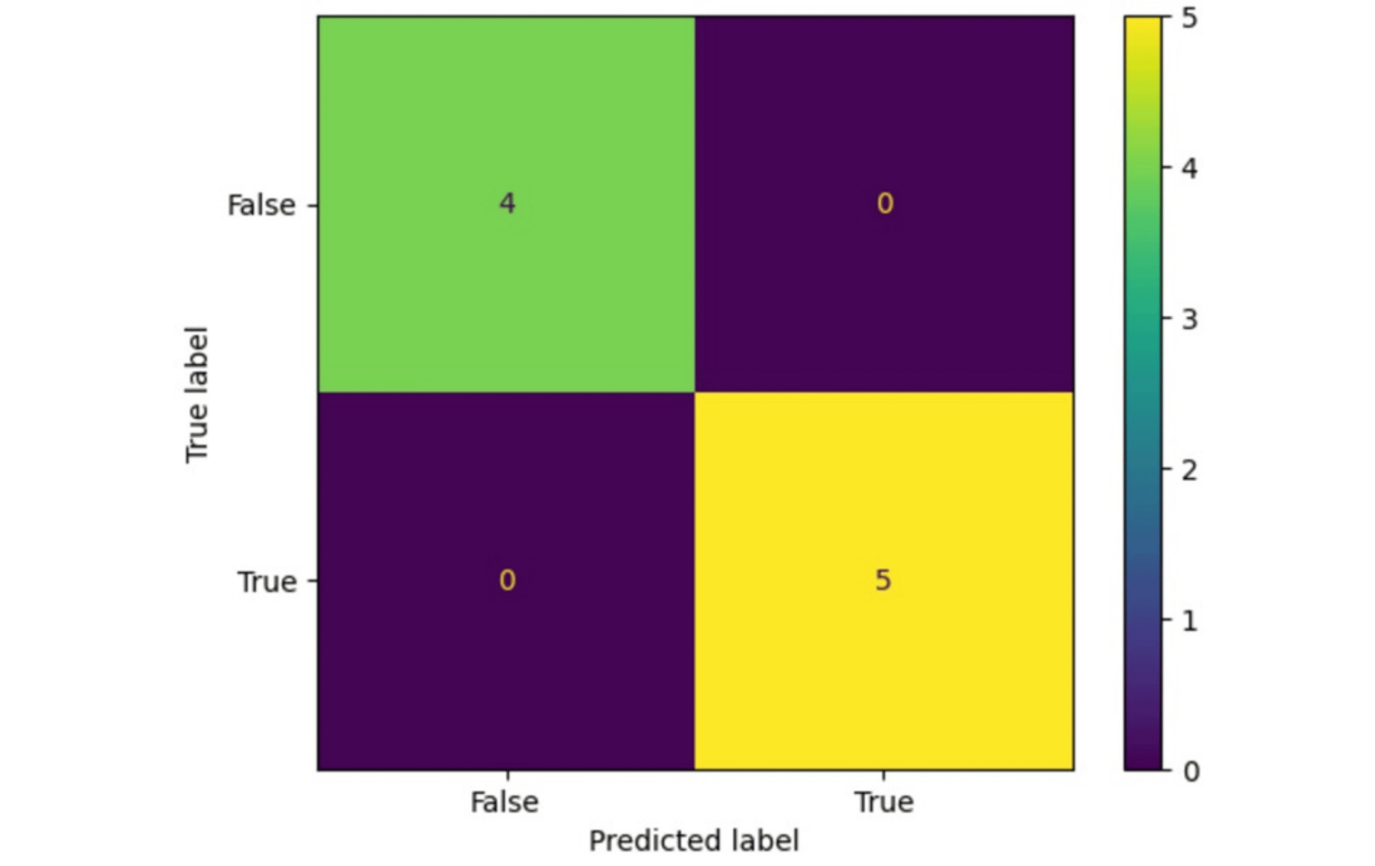
Confusion matrix for DT algorithm (maximum depth = 3), with classification score of 100%

To make sure that the models comply with clinical norms and expectations, we included domain experts and healthcare professionals in the process. Additionally, patient privacy and confidentiality were of top priority, and the ethical implications of dealing with healthcare data were determined. A confusion matrix was employed, which is a valuable instrument for assessing a classification model's efficacy, particularly when applied to medical issues (Figure 5). It offers an in-depth analysis of how the model's predictions compare to the real ground truth. It is critical to comprehend the kinds of mistakes the model produces, particularly in the medical domain, where accurate forecasts are vital [67-69].

## Discussion

Our study analyzed the cell count baseline during treatment to investigate different hematologic toxicities during chemoradiation therapy for cervical cancer. The distribution of the severity and occurrence of hematologic toxicities varies. Of the toxicities, anemia, and lymphopenia were both prevalent and substantially linked to worse results, but thrombocytopenia and neutropenia were neither common nor predictive. Collectively, these findings underline the significance of additional concurrent toxicities while highlighting the role of lymphocyte and erythrocyte function in increasing responsiveness to anticancer treatment [14].

One typical issue with pelvic chemoradiotherapy that restricts treatment intensity is acute hematologic damage [11].

The overall and age-specific distribution of cervical cancer cases is influenced by early identification through screenings, HPV vaccination, and lifestyle choices. For women to manage their unique risk factors, it is critical that they adhere to screening recommendations and seek advice from medical professionals [70,71].

It has previously been determined that low lymphocyte counts after therapy are a poor prognostic factor for a number of malignancies, including cervical cancer [72-75].

Using a comparable standardized method, it was discovered that grade 4 lymphopenia was significantly correlated with survival in cervical cancer, even after adjusting for patient characteristics and treatment variables such as the total dosage of cisplatin [14].

Our findings further reinforce the notion that good treatment outcomes in cervical cancer are contingent upon a sufficient immune response, including lymphocytes from the patient's immune system, given the mounting data linking severe therapy-related lymphopenia to a poor prognosis [14].

How, exactly, lymphopenia affects cancer survival rates is still unknown. Even after considering functional status or infection-related mortality, several studies have demonstrated that lymphopenia independently predicts poorer cancer survival. This suggests that those with low overall health status may be more susceptible to developing lymphopenia [76,77].

We hypothesize that women who better preserve lymphocyte counts during therapy are able to generate a more powerful immune response to carcinogenesis, hence increasing survival, based on our analysis of lymphopenia and cervical cancer outcomes [14]. 

Our study has several limitations, the most significant being the inclusion of only patients with stage III cervical cancer. This selection limits the generalizability of our findings to patients with other stages of the disease. Future studies should consider including patients across all cancer stages to provide a more comprehensive understanding of treatment efficacy and patient outcomes in cervical cancer.

Prior research has demonstrated racial disparities in hematologic toxicities during therapy, which have impacted survival rates [78,79]. A higher body mass index (BMI) was linked to a lower risk of developing severe lymphopenia. The use of systemic therapy for multiple sclerosis has been linked to this connection [80]. A higher BMI has been linked to a lower risk of developing sarcopenic syndrome, which is characterized by a high neutrophil-to-lymphocyte ratio [81]. A bigger bone marrow volume, which has been demonstrated to be protective against hematologic toxicities after pelvic radiation, may be the cause of a higher BMI [82].

It is generally known that there is a high association between anemia and tumor recurrence in individuals with cervical cancer, with hypoxia-induced radiation resistance being the most often mentioned cause. According to several studies, anemia before, during, or after therapy is indicative of a bad prognosis [83].

Cervical cancer is a major worldwide burden and continues to pose a considerable therapeutic challenge, particularly in low- and middle-income countries (LMICs) where resources are scarce and available treatment choices are frequently inaccessible [84]. Therefore, it is imperative that all nations support the 2020 World Health Assembly resolution calling for the "elimination of cervical cancer" by 2030 through the accomplishment of the following three goals: (1) 90% of girls immunized against HPV by the age of 15; (2) high-performance test screening for 70% of women at 35 and 45 years old; and (3) 90% of precancerous lesions treated and 90% of invasive cancer cases managed [85-88].

Despite the great progress in using combination medicines to boost the effectiveness of single-agent treatments, there is still a great need for new and better therapeutics to treat cervical cancer. Moreover, there is a connection between the incapacitating side effects and tumor medication resistance of the available cervical cancer treatments [89,90].

The overall and age-specific distribution of cervical cancer cases is influenced by early identification through screenings, HPV vaccination, and lifestyle choices. For women to manage their unique risk factors, it is critical that they adhere to screening recommendations and seek advice from medical professionals [91].

## Conclusions

Standard machine learning techniques, including naïve Bayes, random forest, decision trees, and TabPFN, can successfully improve the diagnostic accuracy of cervical cancer. By leveraging these algorithms, we can enhance prediction models and identify key predictive markers, contributing to more effective early detection and personalized treatment strategies for cervical cancer.

Artificial intelligence enhances the selection of chemotherapy for cervical cancer by integrating patterns identified in laboratory analyses, such as hematological parameters. This allows clinicians to choose the most effective and personalized treatment options with greater precision and reduced side effects. According to our study, carboplatin could be a better drug if palliation is the aim, as it is linked to reduced toxicity.
